# Comparative longitudinal variation of total IgG and IgA anti-SARS-CoV-2 antibodies in recipients of BNT162b2 vaccination

**DOI:** 10.1515/almed-2021-0086

**Published:** 2021-12-20

**Authors:** Giuseppe Lippi, Gian Luca Salvagno, Brandon M. Henry, Laura Pighi, Simone De Nitto, Gianluca Gianfilippi

**Affiliations:** Section of Clinical Biochemistry, University of Verona, Verona, Italy; Service of Laboratory Medicine, Pederzoli Hospital, Peschiera del Garda, Italy; Clinical Laboratory, Division of Nephrology and Hypertension, Cincinnati Children’s Hospital Medical Center, Cincinnati, OH, USA; Disease Intervention & Prevention and Population Health Programs, Texas Biomedical Research Institute, San Antonio, TX, USA; Medical Direction, Pederzoli Hospital, Peschiera del Garda, Italy

**Keywords:** antibodies, COVID-19, immune response, SARS-CoV-2, vaccination

## Abstract

**Objectives:**

This article aims to summarize the 6-month variation of a vast array of anti-SARS-CoV-2 antibodies in recipients of BNT162b2 mRNA-based vaccination.

**Methods:**

The study population consisted of 84 baseline SARS-CoV-2 seronegative healthcare employees (median age 45 years, 53.6% females), receiving mRNA-based BNT162b2 primary vaccination cycle. Blood was collected before the first and second BNT162b2 vaccine doses, as well as 1, 3 and 6 months afterwards. The serum titers of the following anti-SARS-CoV-2 antibodies were assayed: total anti-RBD (receptor binding domain), anti-spike trimeric IgG, anti-RBD IgG and anti-spike S1 IgA.

**Results:**

All antibodies’ levels peaked 1 month after vaccination, but then displayed a considerable decrease. The median rates of 6-month decline were −95% for IgG anti-SARS-CoV-2 RBD, −85% for IgG anti-SARS-CoV-2 trimeric spike, −73% for IgA anti-SARS-CoV-2 S1 and −56% for total anti-SARS-CoV-2 RBD antibodies, respectively. The median time of seronegativization was estimated at 579 days for total anti-SARS-CoV-2 RBD antibodies, 271 days for IgG anti-SARS-CoV-2 trimeric spike, 264 days for IgG anti-SARS-CoV-2 RBD and 208 days for IgA anti-SARS-CoV-2 S1, respectively. The rate of seropositive subjects declined from 98–100% at the peak to 50–100% after 6 months. The inter-individual variation of anti-SARS-CoV-2 antibodies reduction at 6 months was 3–44% from the peak.

**Conclusions:**

The results of this longitudinal serosurvey demonstrate that the titer of anti-SARS-CoV-2 antibodies declined 6 months after BNT162b2 vaccination, with median time of IgG/IgA seronegativization estimated between 7 and 9 months, thus supporting the opportunity of administering vaccine boosters approximately 5 to 6 months after the last dose of the primary vaccination cycle.

## Introduction

The ongoing severe acute respiratory syndrome coronavirus 2 (SARS-CoV-2) pandemic outbreak is posing unprecedented challenges to human health, society and economy. With over 5 million deaths throughout November 2021, coronavirus disease 2019 (COVID-19) can now be considered the largest catastrophe involving humanity since the Spanish flu pandemic in 1918–19 [1]. Despite the widespread use of physical preventive measures such as social distancing or isolation, face masking and hand hygiene, has been largely advocated or even mandated in some countries, COVID-19 vaccination seems now the only tangible means for preventing or limiting virus spread [2]. Although several types of COVID-19 vaccines (inactivated, protein-, DNA- or RNA-based) have been developed and approved for use in adults and even in children by many medicines agencies all around the world [3], their efficacy differs widely. In particular, a recent meta-analysis of real-world studies showed that mRNA-based vaccines (e.g., BNT162b2 and mRNA-1273) display better efficacy compared to adenovirus-based (e.g., ChAdOx1 and Ad26.COV2.S) and inactivated (e.g., CoronaVac) vaccines (i.e., 85–100% vs. 65–91% efficacy for preventing symptomatic COVID-19) [4]. Despite such a remarkable COVID-19 vaccine efficacy, considerably higher than that elicited by influenza vaccine against the risk of developing symptomatic infection [5], waning protection has been clearly demonstrated over time, and more specifically 5–6 months after vaccination, which is hence leading the way to reinforced vaccination campaigns based on administration of booster COVID-19 vaccine doses to large parts of the population [6]. Since COVID-19 vaccine efficacy depends largely on presence and persistence of anti-SARS-CoV-2 neutralizing antibodies [7], this article aims to summarize the 6-month variation of a vast array of anti-SARS-CoV-2 antibodies in recipients of BNT162b2 mRNA-based primary vaccination.

## Materials and methods

Our original study population consisted of one hundred SARS-CoV-2 seronegative employees of Hospital of Peschiera del Garda (Italy), who received the mRNA-based Pfizer/BioNTech BNT162b2 vaccine (Comirnaty, Pfizer Inc., New York, US; two 30 µg doses, 21 days apart). Blood samples were collected immediately before administering the first and second vaccines doses, and then 1, 3 and 6 months after the second administration. This study protocol has been comprehensively described in previous reports [8], [9], [10]. Serum was separated by centrifugation at 3,000×*g* for 15 min at room temperature. The serum titers of the following anti-SARS-CoV-2 antibodies were assayed: total anti-RBD antibodies (Roche Elecsys Anti-SARS-CoV-2 S chemiluminescent immunoassay on Roche Cobas 6000; Roche Diagnostics, Basel, Switzerland; positive result: ≥0.82 BAU/mL); anti-spike trimeric IgG (DiaSorin Trimeric spike IgG on Liaison XL; DiaSorin, Saluggia, Italy; ≥33.8 BAU/mL); anti-RBD IgG (ACCESS SARS-COV-2 IgG II on ACCESS 2; Beckman Coulter Inc., Brea, CA, US; positive result: ≥10 AU/mL); and anti-spike S1 IgA (Anti-SARS-CoV-2 ELISA IgA; Euroimmun, Lübeck, Germany; positive result: ≥1.1 ratio). All these tests were shown to display excellent correlation with reference neutralization assays [11], [12], [13]. Test results were expressed with both the measuring unit suggested by the manufacturers (binding antibodies unit [BAU], when available) and as ratio from the baseline (i.e., [Value at time point]/[Value at baseline]). Results were presented as median and interquartile range (IQR). All participants gave a written informed consent for being vaccinated and for participating to the longitudinal serosurvey. The study was carried out according to the Declaration of Helsinki and was approved by the Ethics Committee of the Provinces of Verona and Rovigo (59COVIDCESC; November 3, 2021).

## Results

**Figure 1:**
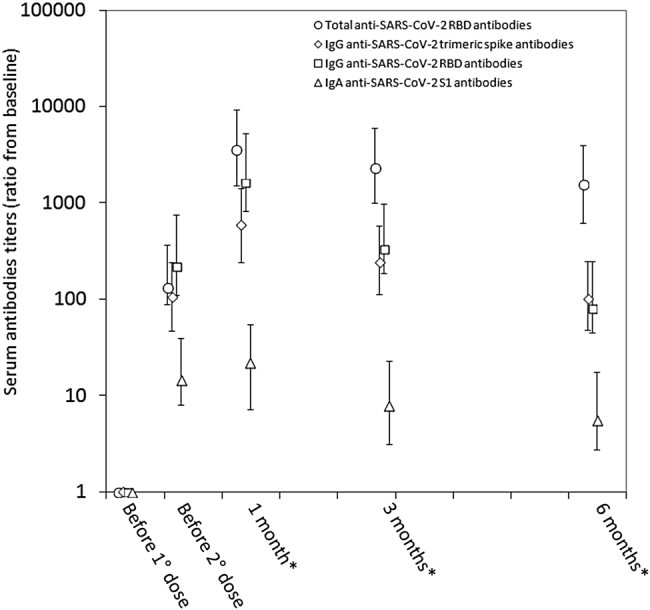
Kinetics of total anti-RBD (receptor binding domain), anti-spike trimeric IgG, anti-RBD IgG and anti-spike S1 IgA serum antibodies levels in seronegative recipients of BNT162b2 mRNA-based vaccination. *After the second vaccine dose.

**Figure 2:**
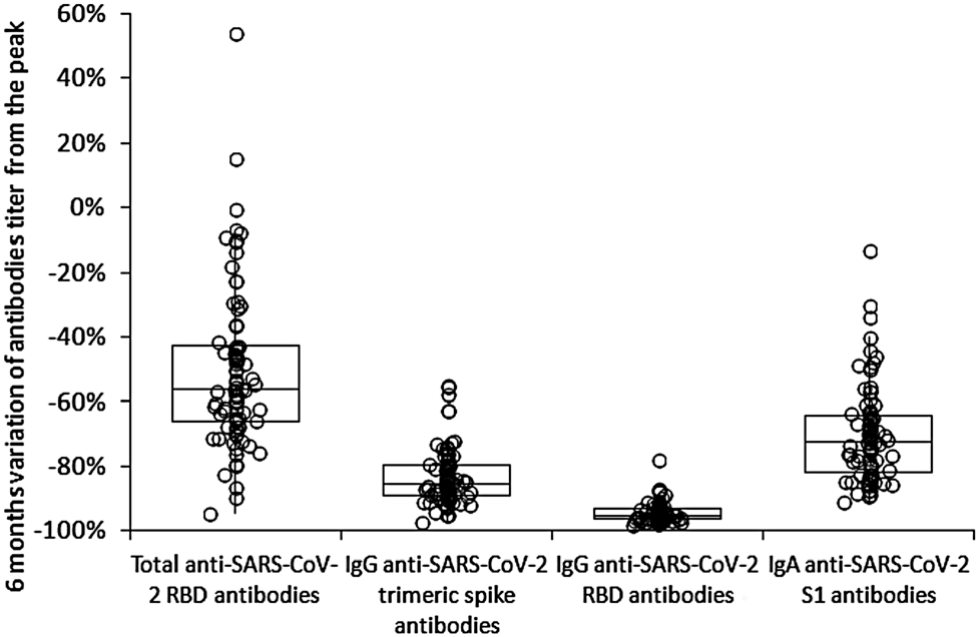
Percentage 6-month reduction from the peak of total anti-RBD (receptor binding domain), anti-spike trimeric IgG, anti-RBD IgG and anti-spike S1 IgA serum antibodies levels in seronegative recipients of BNT162b2 mRNA-based vaccination.

**Figure 3:**
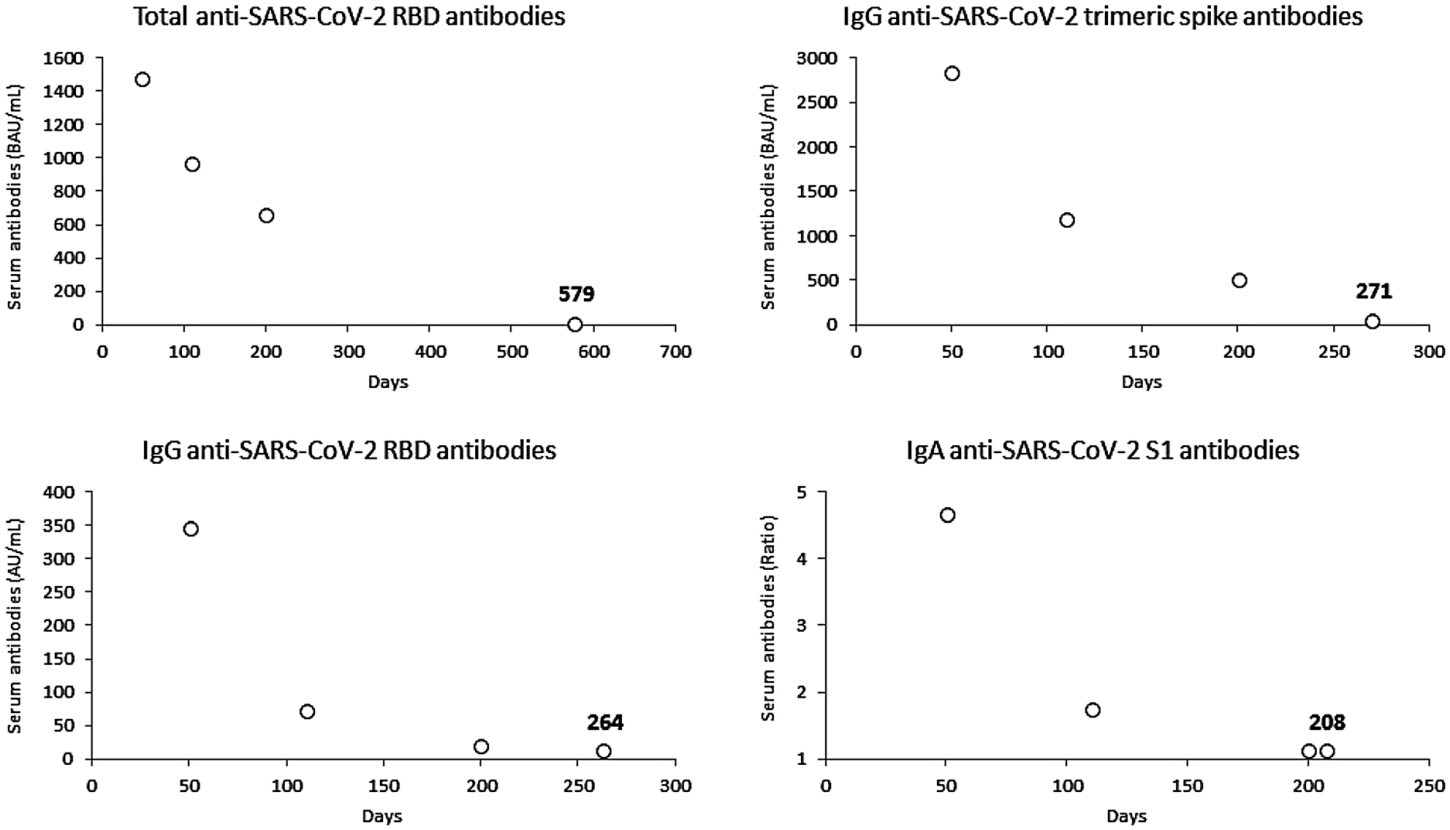
Estimated median time of seronegativization from the peak of total anti-RBD (receptor binding domain), anti-spike trimeric IgG, anti-RBD IgG and anti-spike S1 IgA serum antibodies levels in seronegative recipients of BNT162b2 mRNA-based vaccination.

The final sample consisted of 84 baseline SARS-CoV-2 seronegative healthcare employees (median age 45 years, IQR 31–53 years; 53.6% females), as 16 subjects were lost during follow-up. The variation of the different antibodies tested throughout this post-vaccine 6-month longitudinal serosurvey is shown in Figure 1. The peak of all antibodies was reached 1 month after vaccination, and the ratio from baseline was the highest for total anti-SARS-CoV-2 RBD antibodies (median ratio, 3,550; IQR, 2,040–5,682), followed by IgG anti-SARS-CoV-2 RBD antibodies (median ratio, 1,620; IQR, 795–3,618), IgG anti-SARS-CoV-2 trimeric spike antibodies (median ratio, 584; IQR, 342–834) and IgA anti-SARS-CoV-2 S1 antibodies (median ratio, 22; IQR, 15–32). A notable decrease was then recorded, with median rates of 6-month decline of the antibodies ratio from the peak levels (i.e., [6-month value]/[Baseline value]) decreasing by −95% (IQR, −93% to −96%) for IgG anti-SARS-CoV-2 RBD antibodies, −85% (IQR, −80% to −89%) for IgG anti-SARS-CoV-2 trimeric spike antibodies, −73% (IQR, −64% to −81%) for IgA anti-SARS-CoV-2 S1 antibodies, and −56% (IQR, −43% to −66%) for total anti-SARS-CoV-2 RBD antibodies (Figure 2). According to these trends, we estimated by logarithmic fit that the median time of seronegativization could be 579 days for total anti-SARS-CoV-2 RBD antibodies, 271 days for IgG anti-SARS-CoV-2 trimeric spike, 264 days for IgG anti-SARS-CoV-2 RBD and 208 days for IgA anti-SARS-CoV-2 S1, respectively (Figure 3).

**Figure 4:**
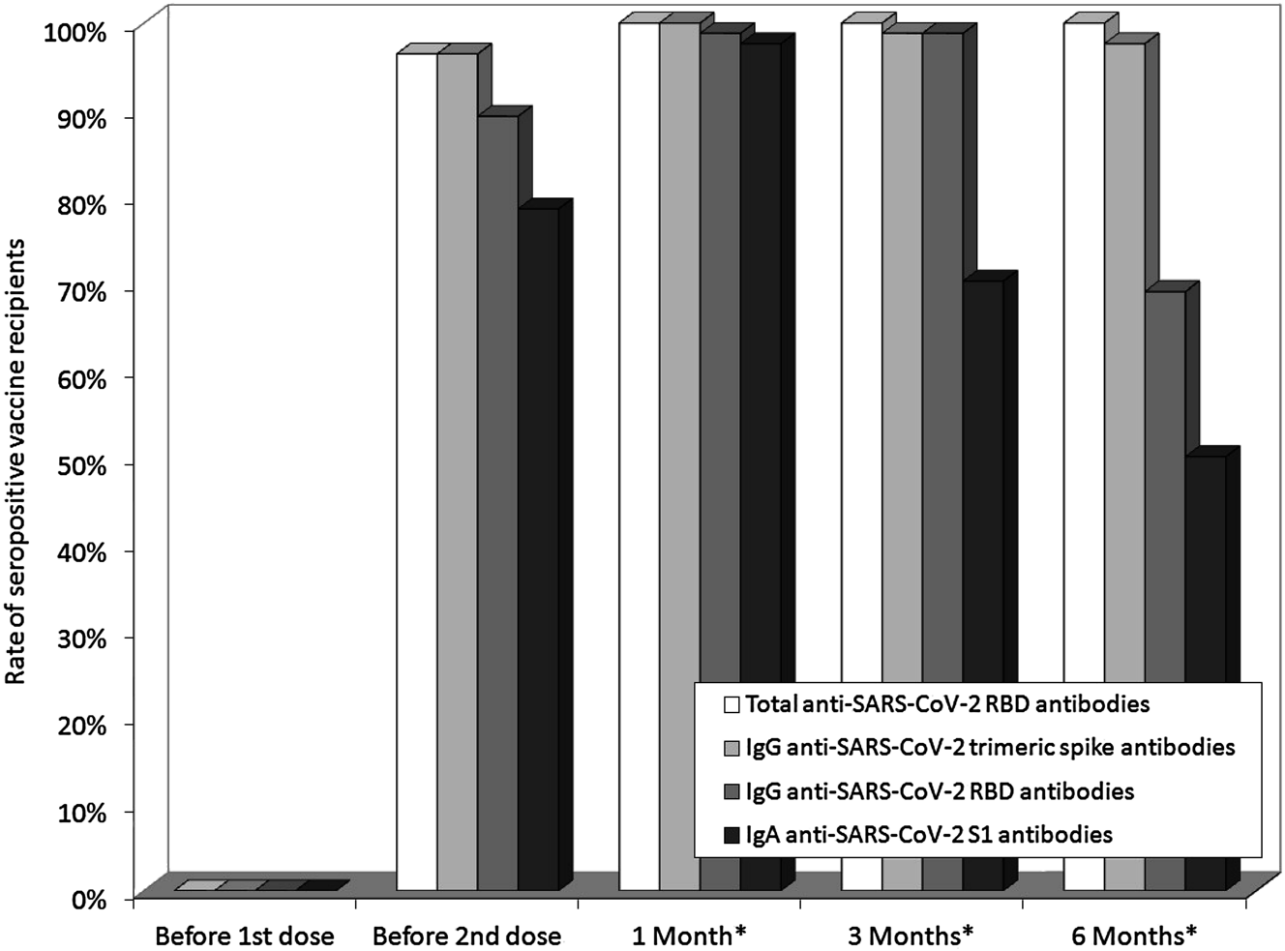
Rate of total anti-RBD (receptor binding domain), anti-spike trimeric IgG, anti-RBD IgG and anti-spike S1 IgA seronopositive recipients of BNT162b2 mRNA-based vaccination. *After the second vaccine dose.

The rate of seropositive subjects found throughout the study period is summarized in Figure 4, increasing from 0% to 73–96% and 98–100% after the first and second BNT162b2 vaccine doses, slightly decreasing to 70–100% at 3 months after the second BNT162b2 vaccine dose, and finally to 50–100% at 6 months after the second vaccine dose. Specifically, IgA anti-SARS-CoV-2 S1 displayed the largest seronegativization (i.e., 50%), whilst all subjects were still positive for total anti-SARS-CoV-2 RBD antibodies after 6 months. The coefficient of variation of the reduction from the peak recorded at 6 months after the second vaccine dose was 3% for IgG anti-SARS-CoV-2 RBD antibodies, 10% IgG anti-SARS-CoV-2 trimeric spike antibodies, 20% for IgA anti-SARS-CoV-2 S1 antibodies, but as high as 44% for total anti-SARS-CoV-2 RBD antibodies, respectively.

## Discussion

The results which have emerged from this longitudinal serosurvey demonstrate that the median titer of a vast array of anti-SARS-CoV-2 antibodies significantly declined 6 months after administration of the second BNT162b2 vaccine dose of the primary cycle, with half of vaccine recipients already becoming IgA anti-SARS-CoV-2 S1 seronegative. A significant number of subjects also became anti-SARS-CoV-2 RBD IgG seronegative (∼30%), whilst the majority (i.e., >98%) maintained titers of anti-SARS-CoV-2 trimeric spike IgG and total anti-SARS-CoV-2 RBD antibodies above the respective positivity thresholds.

The combination of the longitudinal anti-SARS-CoV-2 antibodies variations up to 6 months after completing BNT162b2 primary vaccination cycle in our population leads the way to some biological and clinical reflections. Recent data describing the efficacy of COVID-19 mRNA vaccines (i.e., BNT162b2 and mRNA-1273) over time have clearly reported worrying figures concerning the protection against any type of SARS-CoV-2 infection, with values decreasing from 87–89% to 43–58% at 6 months after vaccination [14], while the effectiveness of these same vaccines against hospitalization seemingly remained stable throughout the same period (i.e., from 88–93% to 77–92%) [15]. Since vaccine efficacy appears largely dependent on anti-SARS-CoV-2 neutralizing antibodies titer [7], the sharpest decline and larger seronegativization of IgA anti-SARS-CoV-2 S1 observed in our population of healthcare workers precisely mirrors the considerable decrease of protection against the risk of developing any type of SARS-CoV-2 infection (either asymptomatic or symptomatic) seen in recent epidemiological studies, whereby this class of antibodies constitutes the primary mucosal defense against many infectious (especially respiratory) diseases [16, 17]. Conversely, the relatively lower reduction of COVID-19 vaccine efficacy against symptomatic and especially severe SARS-CoV-2 infections seems to be mirrored by the still valid immunity secured by total anti-SARS-CoV-2 RBD and IgG anti-SARS-CoV-2 trimeric spike antibodies, as seen in or study, and summarized in Figure 3. This aspect, coupled with development and persistence of memory and cell-mediated immunity after COVID-19 vaccination, may represent suitable protection for preventing both local (i.e., nasopharyngeal, pulmonary) and systemic viral spread [18, 19]. Nonetheless, it is not either reassuring that the serum levels of these two antibodies classes displayed a considerable and progressive reduction from the peak (i.e., −56% and −73%, respectively), which suggest that the levels of these immunoglobulins may also decline below the positivity threshold in a relatively short period, that we estimated at around 9–19 months.

The broad inter-individual variation in the rate of decay of IgA anti-SARS-CoV-2 S1 and total anti-SARS-CoV-2 RBD antibodies found in our population (i.e., 20% and 44%, respectively) is another important aspect, which underpins and confirms the importance of serological monitoring post-vaccination [20]. This practice may in fact enable to timely identify subjects with faster and sharper decline of serum antibodies titer [21], in whom booster doses of COVID-19 vaccines should be prioritized, especially for restoring a sufficient level of neutralization against highly mutated SARS-CoV-2 lineages (e.g., Omicron B.1.1.529) [22]. Our data on median seronegativization time are also perfectly aligned with the recent recommendations of the Advisory Committee on Immunization Practices’ for additional booster doses of COVID-19 vaccines [22]. In fact, besides total anti-SARS-CoV-2 RBD antibodies, whose negativization would probably occur after 19 months, the median seronegativization times of all the other antibodies classes tested in our study were comprised between 7 and 9 months (Figure 3), thus strongly endorsing the use of additional COVID-19 vaccine boosters 5 to 6 months after the last vaccine dose, especially in more vulnerable populations.
